# Physiological adaptations of active postmenopausal women and matched men to the multi-day Sardinia Selvaggio Blu wild trek: a gender-comparative pilot study

**DOI:** 10.1007/s00421-023-05330-0

**Published:** 2023-10-11

**Authors:** Marco Morrone, Anna Boi, Martina Meloni, Gianluca Martinez, Lucia Ventura, Maurizio Pusole, Giovanna Ghiani, Antonio Crisafulli, Pasquale Bandiera, Lucia Cugusi, Franca Deriu, Andrea Manca

**Affiliations:** 1 Department of Biomedical Sciences, University of Sassari, Viale S. Pietro 43/B, 07100 Sassari, Italy; 2 Sports Physiology Lab, Department of Medical Sciences and Public Health, University of Cagliari, Cagliari, Italy; 3 Unit of Endocrinology, Nutrition, and Metabolic Disorders, AOUSS, Sassari, Italy

**Keywords:** Body composition, Energy expenditure, Cardiorespiratory fitness, Hiking, Trekking

## Abstract

**Purpose:**

To evaluate the effects of wild trekking by examining, in postmenopausal women, the physiological adaptations to an intensive 5-day wild trek and comparing their responses to those displayed by a group of men of comparable age, training status and mountaineering skills.

**Methods:**

Six healthy, active postmenopausal women in their sixth decade of life participated in the study. Six men of comparable age and training status were also enrolled for gender-based comparisons. The participants traversed the Selvaggio Blu wild trek (Sardinia, Italy) completing a total of 56 km, for an overall height differential of 14,301 m. During all 5-day trek, subjects were supervised by two alpine guides. Changes in body composition, cardiorespiratory fitness, and metabolic patterns of energy expenditure were evaluated before and after the intervention.

**Results:**

Total energy expenditure during the trek was significantly higher (*p* = 0.03) in women (12.88 ± 3.37 kcal/h/kg) than men (9.27 ± 0.89 kcal/h/kg). Extracellular (ECW) and intracellular water (ICW) increased significantly following the trek only in women (ECW: − 3.8%; *p* = 0.01; ICW: + 3.4%; *p* = 0.01). The same applied to fat-free mass (+ 5.6%; *p* = 0.006), fat mass (− 20.4%; *p* = 0.006), skeletal muscle mass (+ 9.5%; *p* = 0.007), and appendicular muscle mass (+ 7.3%; *p* = 0.002). Peak *V*O_2_/kg (+ 9.4%; *p* = 0.05) and fat oxidation (at 80 W: + 26.96%; *p* = 0.04; at 100 W: + 40.95%; *p* = 0.02; at 120 W: + 83.02%; *p* = 0.01) were found increased only in women, although no concurrent changes in partial pressure of end-tidal CO_2_ (PETCO_2_) was observed.

**Conclusions:**

In postmenopausal women, a 5-day, intensive and physically/technically demanding outdoor trekking activity led to significant and potentially relevant changes in body composition, energy balance and metabolism that are generally attained following quite longer periods of training.

## Introduction

The outdoor environment is increasingly being used as a setting for exercise-based therapeutic and training/reconditioning purposes, with many organizations and agencies recommending wilderness experiences to attain sociological, psychological, and physiological benefits (Kelley 1993). Among the broad spectrum of outdoor activities, hiking and trekking have emerged as popular recreational activities that provide several health benefits including reduced blood pressure, body fat, risk of heart disease and mortality along with increased cardiorespiratory fitness, bone and muscle mass (Mitten et al. 2016). Trekking differs from hiking in that it involves mountaineering in wild natural environment for multiple days. Depending on personal preferences, physical conditions, technical skills and experience, trekking can range from a leisurely, low-intensity activity to a strenuous, high-intensity training (Hawke and Jensen 2020). While the physiological and health-related effects of hiking and trekking have been the topic of a relatively large body of literature both in healthy and diseased populations, less is known about the employment of these interventions in postmenopausal women.

Due to the consistently low circulating estradiol and elevated gonadotropins, postmenopausal women face a constellation of physical changes and major health concerns (Chae and Derby 2011). Among these, the increased prevalence in cardiovascular diseases, the decrease in endothelial function and high-density lipoprotein cholesterol levels have emerged as prominent features. Osteoporosis and loss of muscle and bone mass are other serious issues that postmenopausal women must face (Camacho et al. 2016). Mood and cognitive alterations also occur in aging women because of estrogen loss, making it more difficult for postmenopausal women to maintain an active lifestyle (Kawas et al. 1997). Some of the abovementioned changes and health concerns can be mitigated by contrasting sedentarism and engaging both in aerobic and resistance trainings exercise (Asikainen et al. 2004). In this regard, trekking intrinsically incorporates elements of muscle strength and endurance, bone loading, balance, dexterity, coordination, physical awareness, and mountaineering skills that are specifically relevant for postmenopausal women’s health to contrast age-related morphological and functional losses.

To the best of our knowledge, the effects of outdoor trekking on postmenopausal women’s health and functioning have been poorly investigated. Following 12 weeks of moderate-intensity trekking, thirty-two postmenopausal obese women displayed increased cardiovascular health (body mass index, BMI, body fat, blood pressure, glucose, triglycerides, and total cholesterol) and fitness (muscle strength, muscle endurance, balance, and flexibility), as compared to a group of control subjects undergoing no exercise (Kang 2014). However, no data are yet available on the acute effects, i.e., the physiological adaptations of postmenopausal women to trekking activities. Based on the above rationale, the main purpose of this study was to evaluate the health potential of trekking by (1) examining, in active postmenopausal women with mountaineering expertise, the physiological adaptations to an intensive and challenging 5-day trekking experience, and (2) assessing if these responses were different from those displayed by a group of male participants of comparable age, training status and mountaineering skills.

## Methods

### Design and participants

The study design was set as a gender-comparative, interventional pilot study. Six healthy, active postmenopausal women in their sixth decade of life (51–60 years old) were recruited for this study after preliminary anamnestic and clinical examinations performed by a physician. The eligibility requirements were as follows: (1) healthy individuals with no documented cardiovascular, respiratory, neurological, or musculoskeletal conditions; (2) active fitness status; (3) consolidated trekking experience and rope and/or rappelling dexterity in outdoor environments. Six male volunteers of comparable age, technical and training status were also enrolled for gender-based comparisons. All participants were informed on the aims of the study and its procedures before enrollment, and written informed consent was obtained from all subjects. The study was approved by the Institutional Review Board of the Department of Biomedical Sciences at the University of Sassari (Registration No.: MEDF-3/2022) and was performed in accordance with the Declaration of Helsinki.

### Assessment procedures

All subjects deemed eligible for enrollment underwent a comprehensive set of assessments to characterize three domains, including body composition, cardiorespiratory fitness, and metabolic patterns of energy expenditure. All the tests were administered prior to and 2 days after the 5-day trekking experience to capture any possible acute adaptations.

### Trek monitoring

The participants wore an activity monitor (wGT3X-BT, Actigraph, Pensacola, FL, USA) and a heart rate monitor (H10 Polar Electro Oy, Kempele, Finland) to estimate the energy expenditure to sustain the trek and its intensity.

### Body composition

The participants’ weight and height were determined using a stadiometer (Bilance Salus, Milan, Italy). Total and regional body composition was determined through a bioelectrical impedance vector analysis (BIVA) (BIA 101 BIVA^®^ PRO, Akern, Florence, Italy). The subjects were tested supine, after a 5-min rest, on a non-conducting surface with their upper and lower limbs slightly abducted.

### Cardiorespiratory fitness

All subjects completed an incremental cardiopulmonary exercise test (CPET) under the supervision of a sports medicine specialist (A.C.) on an electromagnetically braked cycle ergometer (Cardioline STS400, S. Pedrino di Vignate, Italy). The CPET was performed utilizing a ramp protocol that involved raising the work rate (WR) every minute. Men began at 30 W with increments of 30 W, while women began at 20 W with increments of 20 W. Gas exchanges and ventilatory parameters were collected breath-by-breath during all trials by means of a portable (mass 400 g) metabolic system (METAMAX^®^3B, CORTEX Biophysik, Leipzig, Germany). The gas analyser was calibrated before each test with ambient air (O_2_ 20.93% and CO_2_ 0.03%) and a gas mixture of known composition (O_2_ 16.00% and CO_2_ 5.00%). Facemasks allowed subjects to simultaneously breathe with mouth and nose, for more comfort. The facemask (Hans Rudolph Inc, Dallas, USA) was equipped with a low-resistance, bidirectional digital turbine (diameter 28 mm) that was calibrated before each test with a 3-L syringe. Heart rate (HR) was monitored continuously using a wireless Polar monitoring system (Polar Electro Oy, Kempele, Finland) synchronized with the metabolic system. The first and second ventilatory thresholds (Vt_1_ and Vt_2_, respectively), the peak aerobic capacity (peak *V*O_2_) and ventilation (peak *V*′*E*), and partial pressure of end-tidal carbon dioxide (PETCO_2_) were determined. The latter was measured along with the respiratory exchange ratio to inform about the rate of utilization of energy substrates (lipids and carbohydrates) at the different work rates of the incremental CPET.

### Nutritional and metabolic profile

All individuals were interviewed and evaluated by a nutrition consultant (G.G.) to create a personalized meal plan for the 5-day trek, during which they were required to consume an energy intake equal to two times their basal metabolic rate. In order to obtain all this information, participants were asked to fill a questionnaire about their eating habits (Turconi et al. 2008) and lifestyle (Mannocci et al. 2010).

Before and after the 5-day trek, energy substrate utilization patterns were evaluated by the same metabolic system employed for the CPET using the respiratory exchange ratio to estimate fat and carbohydrate contribution to energy expenditure to sustain the incremental test.

### Intervention

The participants traversed the Selvaggio Blu (Wild Blue) wild trek in the Baunei district (Sardinia, Italy). Throughout their 5-day trek, the hikers encountered trails of varying degrees of difficulty, as classified by the Italian Alpine Club’s “CAI Scale”, which ranks the trails in terms of difficulty, equipment requirements, and the difficulty of any potential via ferrata (https://www.cai.it/wp-content/uploads/2021/12/allegato-circolare-22_2021-Classificazione-difficolt%C3%A0.pdf). In this variant of the Selvaggio Blu, participants were required to traverse trails at a degree of difficulty between “experienced excursionist” and “moderately difficult” via ferrata (EEA-PD+) levels, including canals, fireplaces, and a few short vertical sections, with the assistance of chains, cables, pegs, and metallic ladders. They traversed a total of 56 km, ascending 7142 m and descending 7159 m for an overall height differential of 14,301 m. During all 5-day trek, subjects were supervised by two alpine guides.

### Statistical analysis

Data analysis was performed using the SPSS software for Windows, version 26.0 (IBM, New York). The Kolmogorov–Smirnov test was employed to assess the normality of data distribution. To monitor gender differences in energy expenditure and daily nutritional energy intake during the trek, a one-way analysis of variance (ANOVA) was performed. Due to the expected differences in cardiorespiratory performance between women and men, to test changes from baseline in the outcomes considered a repeated-measures analysis of covariance (RM-ANCOVA) was performed having fitness baseline (peak *V*O_2_/kg) as covariate and TIME (PRE vs. POST) as within-subjects factor. Bonferroni-adjusted pairwise comparisons were then run to test any changes from pre- to post-intervention within each group. For all comparisons, statistical significance was set at the conventional level of *p* < 0.05.

To quantify the intervention-induced changes in the outcome of interest, the magnitude of the difference was quantified as the standardized effect size (Cohen’s *d*), i.e., the contrast in standard deviations (SDs) between PRE and POST for each group. Effect size interpretation was made considering a change ≤ 0.4 SDs as small, 0.41–0.79 as moderate, ≥ 0.8 as large (Cohen 1988).

## Results

Complete data were obtained from 12 volunteers of comparable age (women: 56.0 ± 3.1 years; 95% CI 52.75–59.25; men: 56.8 ± 4.9 years; 95% CI 51.67–61.99; *p* = 0.73). Tables 1 and 2 report the main findings of the study.

**Table 1:** Body composition data before (pre) and after (post) the 5-day trek experience

Variable (unit of measurement)	Sex	Pre	Post	Statistics^#^
Weight (kg)	Women	54.53 ± 3.48 (48.11, 60.96)	54.28 ± 3.67 (47.44, 61.12)	*p* = 0.04; *d* = 0.58
	Men	79.00 ± 9.36 (72.58, 84.42)	78.00 ± 9.98 (71.96, 85.64)	*p* = 0.31; *d* = 0.16
BMI (kg/m^2^)	Women	21.95 ± 0.67 (19.66, 24.24)	21.85 ± 0.76 (19.58, 24.12)	*p* = 0.04; *d* = 0.60
	Men	26.78 ± 3.50 (24.49, 29.08)	26.67 ± 3.45 (24.39, 28.94)	*p* = 0.37; *d* = 0.14
TBW (%)	Women	56.85 ± 3.31 (54.36, 59.34)	59.38 ± 2.96 (56.49, 62.28)	*p* = 0.007; *d* = 0.88
	Men	59.08 ± 2.01 (56.59, 61.58)	60.57 ± 3.39 (57.67, 63.46)	*p* = 0.45; *d* = 0.11
ECW (%)	Women	48.03 ± 1.68 (46.08, 49.99)	46.33 ± 1.11 (44.61, 48.06)	*p* = 0.01; *d* = 0.83
	Men	46.07 ± 2.53 (44.12, 48.02)	45.33 ± 2.44 (43.61, 47.06)	*p* = 0.30; *d* = 0.16
ICW (%)	Women	51.97 ± 1.68 (50.02, 53.92)	53.67 ± 1.11 (51.94, 55.39)	*p* = 0.01; *d* = 0.83
	Men	53.93 ± 2.53 (51.98, 55.89)	54.67 ± 2.44 (52.94 ± 56.39)	*p* = 0.30; *d* = 0.16
ECW/ICW (% ratio)	Women	0.93 ± 0.06 (0.86, 0.99)	0.86 ± 0.04 (0.81, 0.92)	*p* = 0.01; *d* = 0.84
	Men	0.86 ± 0.09 (0.79, 0.93)	0.83 ± 0.08 (0.77, 0.89)	*p* = 0.31; *d* = 0.16
TBW/BCM (% ratio)	Women	1.11 ± 0.09 (1.05, 1.17)	1.12 ± 0.06 (1.07, 1.17)	*p* = 0.40; *d* = 0.12
	Men	1.11 ± 0.04 (1.05, 1.17)	1.12 ± 0.06 (1.07, 1.17)	*p* = 0.99; *d* = 0.05
ICW/BCM (% ratio)	Women	1.01 ± 0.00 (1.01, 1.02)	1.01 ± 0.00 (1.01, 1.02)	*p* = 0.05; *d* = 0.51
	Men	1.01 ± 0.00 (1.01, 1.02)	1.01 ± 0.00 (1.01, 1.01)	*p* = 0.12; *d* = 0.33
ECW/BCM (% ratio)	Women	0.94 ± 0.06 (0.87, 1.01)	0.88 ± 0.04 (0.81, 0.94)	*p* = 0.01; *d* = 0.83
	Men	0.87 ± 0.09 (0.8, 0.94)	0.841 ± 0.087 (0.78, 0.90)	*p* = 0.30; *d* = 0.16
ICW/FFM (% ratio)	Women	0.67 ± 0.05 (0.64, 0.71)	0.66 ± 0.03 (0.63, 0.69)	*p* = 0.31; *d* = 0.16
	Men	0.67 ± 0.02 (0.64, 0.71)	0.67 ± 0.03 (0.64, 0.7)	*p* = 0.95; *d* = 0.05
FFM (%)	Women	77.63 ± 4.26 (74.35, 80.92)	81.00 ± 3.95 (77.09, 84.91)	*p* = 0.006; *d* = 0.90
	Men	80.27 ± 2.81 (76.98, 83.55)	82.27 ± 4.62 (78.36, 86.17)	*p* = 0.43; *d* = 0.11
FM (%)	Women	22.37 ± 4.26 (19.08, 25.65)	19.00 ± 3.95 (15.09, 22.91)	*p* = 0.006; *d* = 0.90
	Men	19.73 ± 2.81 (16.45, 23.02)	17.73 ± 4.62 (13.83, 21.64)	*p* = 0.43; *d* = 0.11
BCM (%)	Women	51.25 ± 1.79 (49.16, 53.34)	53.03 ± 1.23 (51.17, 54.9)	*p* = 0.01; *d* = 0.82
	Men	53.33 ± 2.72 (51.24, 55.43)	54.15 ± 2.62 (52.29, 56.01)	*p* = 0.28; *d* = 0.18
SMM (%)	Women	34.37 ± 3.24 (31.90, 36.83)	36.88 ± 2.64 (34.05, 39.72)	*p* = 0.007; *d* = 0.88
	Men	40.45 ± 2.04 (37.99, 42.91)	41.933 ± 3.54 (39.1, 44.77)	*p* = 0.46; *d* = 0.11
ASMM (%)	Women	27.91 ± 1.68 (26.61, 29.21)	29.5 ± 1.55 (27.93, 31.07)	*p* = 0.002; *d* = 0.97
	Men	31.88 ± 1.13 (30.57, 33.18)	32.84 ± 1.89 (31.23, 34.41)	*p* = 0.32; *d* = 0.16
BMR (kcal)	Women	1377.85 ± 35.88 (1290.14, 1465.56)	1425.22 ± 39.81 (1344.71, 1505.73)	*p* = 0.001; *d* = 1.0
	Men	1730.38 ± 131.56 (1642.67, 1818.10)	1764.13 ± 118.67 (1683.62, 1844.65)	*p* = 0.06; *d* = 0.48

All data are presented as mean ± standard deviation (SD) and 95% confidence interval (CI)

*kg* kilograms, *BMI* body mass index, *m* meters, *BMR* basal metabolic rate, *Kcal* kilocalories, *TBW* total body water, *ECW* extracellular water, *ICW* intracellular water, *FFM* fat-free mass, *FM* fat mass, *BCM* body cell mass, *SMM* skeletal muscle mass, *ASMM* appendicular skeletal muscle mass

^#^Comparisons by analysis of covariance (ANCOVA) having fitness baseline (*V*O_2_/kg) as the covariate. Adjusted data are not displayed—see Methods). Significance was set for *p* < 0.05

**Table 2:** Cardiorespiratory parameters data before (pre) and after (post) the 5-day trekking experience

Variable (unit of measurement)	Sex	Pre	Post	Statistics^#^
Peak *V*O_2_/kg (ml/kg/min)	Women	34.67 ± 3.67 (26.90, 42.43)	38.83 ± 5.08 (30.85, 46.81)	*p* = 0.05; *d* = 0.53
	Men	48.60 ± 11.93 (40.09, 57.11)	49.60 ± 11.65 (40.86, 58.34)	*p* = 0.48; *d* = 0.10
Peak *V*O_2_/HR (l/min/bpm)	Women	0.22 ± 0.03 (0.16, 0.28)	0.24 ± 0.02 (0.18, 0.3)	*p* = 0.1; *d* = 0.38
	Men	0.31 ± 0.08 (0.25, 0.37)	0.31 ± 0.08 (0.25, 0.37)	*p* = 0.72; *d* = 0.06
Peak *V*′*E* (l/min)	Women	71.50 ± 11.21 (53.97, 89.03)	75.40 ± 14.20 (60.49, 90.31)	*p* = 0.75; *d* = 0.06
	Men	132.16 ± 25.57 (112.96, 151.37)	141.60 ± 18.28 (145.27, 157.93)	*p* = 0.05; *d* = 0.51
Vt_1_ *V*O_2_/kg (ml/kg/min)	Women	15.67 ± 2.25 (12.18, 19.15)	16.33 ± 2.81 (14.18, 21.15)	*p* = 0.62; *d* = 0.07
	Men	17.67 ± 4.93 (14.18, 21.15)	18.33 ± 4.55 (14.90, ‘21.77)	*p* = 0.19; *d* = 0.25
Vt_1_ *V*O_2_/HR (l/min/bpm)	Women	0.16 ± 0.02 (0.11, 0.21)	0.17 ± 0.01 (0.13, 0.22)	*p* = 0.62; *d* = 0.07
	Men	0.20 ± 0.07 (0.15, 0.25)	0.21 ± 0.06 (0.17, 0.25)	*p* = 0.19; *d* = 0.25
Vt_2_ *V*O_2_/kg (ml/kg/min)	Women	29.00 ± 3.29 (19.73, 38.27)	30.83 ± 4.17 (22.54, 39.13)	*p* = 0.58; *d* = 0.08
	Men	38.20 ± 14.60 (28.05, 48.36)	40.60 ± 12.64 (31.514, 49.69)	*p* = 0.03; *d* = 0.67
Vt_2_ *V*O_2_/HR (l/min/bpm)	Women	0.20 ± 0.02 (0.14, 0,27)	0.22 ± 0.02 (0.17, 0.28)	*p* = 0.31; *d* = 0.16
	Men	0.29 ± 0.09 (0.22, 0.35)	0.29 ± 0.07 (0.24, 0.34)	*p* = 0.44; *d* = 0.11

All data are presented as mean ± standard deviation (SD) and 95% confidence interval (CI)

*VO*_*2*_ volume of oxygen consumption, *HR* heart rate, *bpm* beats per minute, *Vt*_*1*_ first ventilatory threshold, *Vt*_*2*_ second ventilatory threshold, *V'E* ventilation, *l* liters, *min* minute

^#^Comparisons by analysis of covariance (ANCOVA) having fitness baseline (*V*O_2_/kg) as the covariate. Adjusted data are not displayed—see Methods). Significance was set for *p* < 0.05

### Energy expenditure during the 5-day trek

One-way ANOVA (factor: SEX) revealed a significant difference (*F*_1,10_ = 6.427; *p* = 0.03) in energy expenditure normalized by skeletal muscle mass (SMM) during the trek between women (12.88 ± 3.37 kcal/h/SMM) and men (9.27 ± 0.89 kcal/h/SMM). Daily nutritional energy intake was 2402 ± 113 kcal for women and 3328 ± 227 kcal for men (*p* < 0.0001).

### Anthropometrics and body composition

Table 1 reports complete data for this domain. RM-ANCOVA (covariate: fitness baseline) revealed a non-significant main effect of TIME (*F*_1,8_ = 3.35; *p* = 0.10) for body weight. However, Bonferroni-adjusted pairwise comparisons detected a significant decrease in body weight only in women (− 1%; *p* = 0.04). Likewise, a decrease in BMI was observed only in women (− 1%; *p* = 0.04).

RM-ANCOVA revealed no significant main effect of TIME (*F*_1,8_ = 2.62; *p* = 0.14) for total body water (TBW). Pairwise comparisons detected a significant increase in TBW only in women (+ 5.8%; *p* = 0.007). Accordingly, significant changes in extracellular water (ECW) (− 3.8%; *p* = 0.01) and intracellular water (ICW) (+ 3.4%; *p* = 0.01) were observed only in women. The ECW to ICW (− 7%; *p* = 0.01) and ECW to BCM (− 7.4%; *p* = 0.01) ratios were significantly decreased at the post only in women.

Adjusted pairwise comparisons showed significant changes only in women for fat-free mass (FFM: + 5.6%; *p* = 0.006), fat mass (FM: − 20.4%; *p* = 0.006), skeletal muscle mass (SMM: + 9.5%; *p* = 0.007), appendicular skeletal muscle mass (ASMM: + 7.3%; *p* = 0.002) and body cellular mass (BCM: + 3.6%; *p* = 0.01).

Only statistical trends towards significance or no significant changes at all were detected in the two sexes for TBW to BCM, ICW to BCM and ICW to FFM ratios, as detailed in Table 1.

**Fig. 1:**
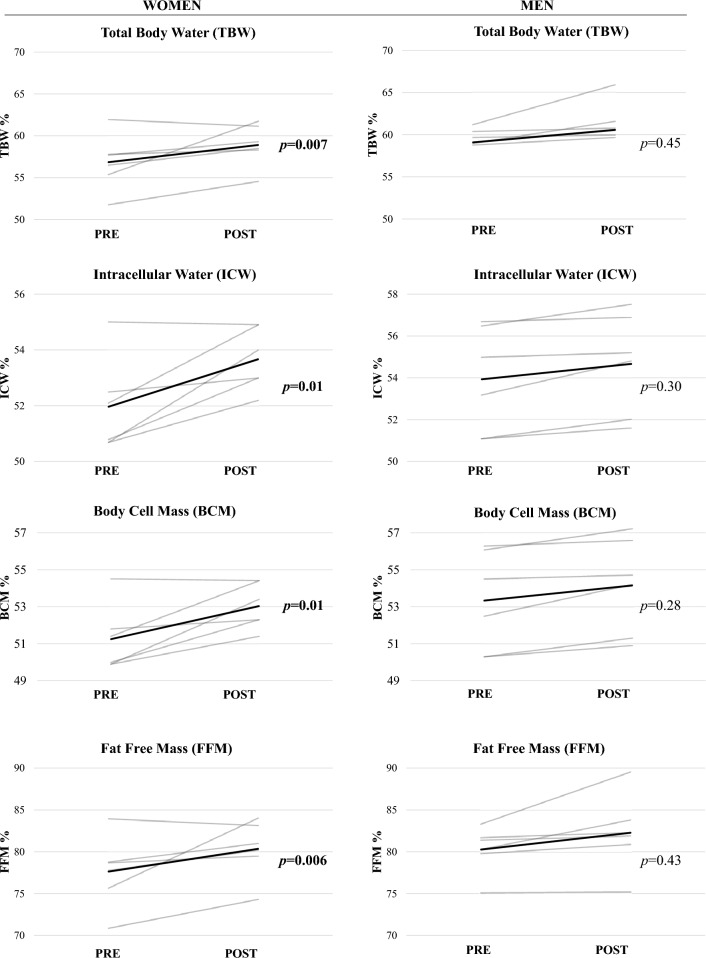

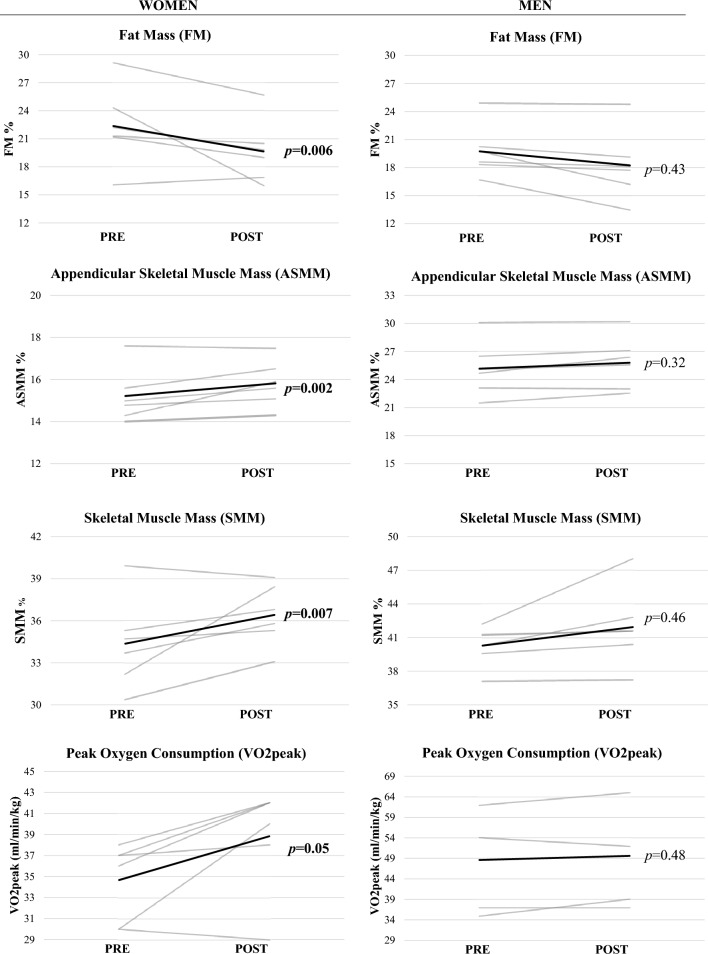
**A**, **B** Results of the individual data analyses on the main outcomes of the study

Figure 1A and B display individual data separately from women and men for those body composition variables for which statistically significant sex-based differences were detected

### Cardiorespiratory fitness

RM-ANCOVA (covariate: fitness baseline) showed no significant main effects of time for *V*O_2_/kg, *V*O_2_/HR at peak, Vt_1_ and Vt_2_ as well as peak *V*′*E*. Table 2 summarizes the results of the Bonferroni-adjusted pairwise comparisons, which revealed significant increases only in women for peak *V*O_2_/kg (+ 9.4%; *p* = 0.05), while men displayed significantly increased *V*O_2_/kg at Vt_2_ (+ 12.7%; *p* = 0.03), as well as increased *V*′*E* at peak *V*O_2_ (+ 10%; *p* = 0.05).

No significant changes were detected for *V*O_2_/HR at peak, Vt_1_ and Vt_2_, nor for *V*O_2_/kg at Vt_1_.

Individual data for peak *V*O_2_/kg are displayed in Fig. 1.

### Metabolic rates and energy substrate utilization

**Fig. 2:**
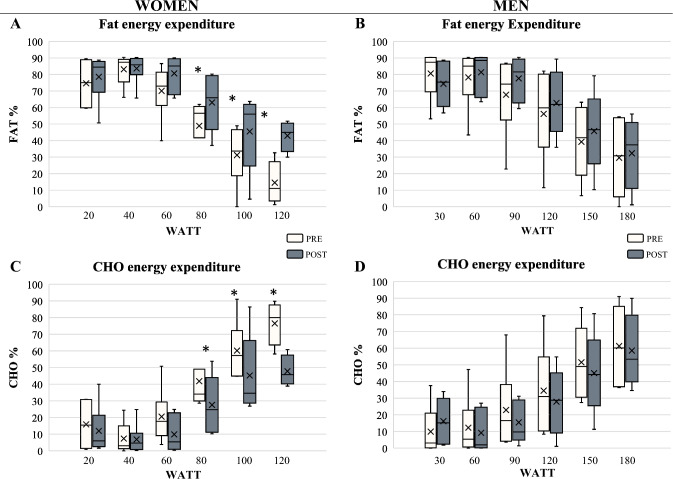
Energy substrate utilization patterns during the incremental cardiopulmonary exercise test. **A** and **B** display the contributions of fat sources in women and men, respectively. **C** and **D** display the contributions of carbohydrate sources

Basal metabolic rate (BMR) was significantly increased only in women (+ 4.1%; *p* = 0.001). Figure 2 displays the results of the gender-based patterns of energy substrate utilization. Following the 5-day trek experience, RM-ANCOVA (covariate: fitness baseline) revealed significant changes in the oxidation of lipids and carbohydrates during CPET. EE derived from fat oxidation (EE%_FAT_) was found significantly increased only in women at 80 W (+ 26.96%; *p* = 0.04; *d* = 0.56), 100 W (+ 40.95%; *p* = 0.02; *d* = 0.70) and 120 W (+ 83.02%; *p* = 0.01; *d* = 0.87). Accordingly, energy expenditure derived from carbohydrate oxidation (EE%_CHO_) was found significantly reduced only in women at 80 W (− 49.6%; *p* = 0.04; *d* = 0.56), 100 W (− 35.4%; *p* = 0.02; *d* = 0.73) and 120 W (− 40.5%; *p* = 0.01; *d* = 0.87).

**Fig. 3:**
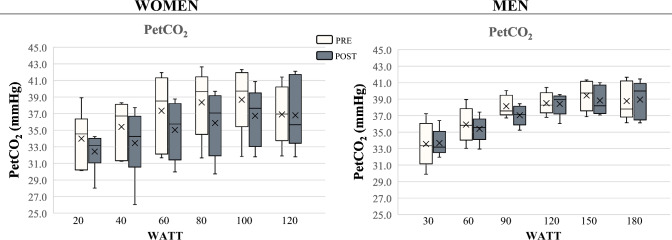
Results of partial pressure of end-tidal carbon dioxide (PETCO_2_) during the incremental cardiopulmonary exercise test

However, when validating these data also assessing PETCO_2_, no changes in this parameter were detected in both sexes at the post-intervention assessment, as displayed in Fig. 3.

## Discussion

The present study examined the physiological adaptations to an intensive and challenging 56-km long, 5-day trekking experience in postmenopausal active women, and assessed if gender-based differences emerged in these adaptations, by comparing them to a group of male participants of comparable age, training status and mountaineering skills. In addition, participants were strictly monitored in terms of fluid and food intake, with a standardized nutritional regimen that had been tailored to each participant according to the basal metabolic rates and anthropometric characteristics evaluated at study entry.

The main finding of this study is that the short-term, intensive, and challenging outdoor activity here completed led to significant improvements in body composition, cellular hydration, cardiorespiratory performance, and metabolic patterns of energy substrate utilization only in women, whereas changes in men’s performance, anthropometric features and metabolic rates only showed trends toward significance. The observed changes were also of potential practical relevance, i.e., capable to make a tangible difference in the participants’ physical performance—according to the large effect sizes here shown, which approached or surpassed the 1-SD of pre-to-post change.

As expected, at baseline men and women exhibited the well-known differences in maximal aerobic and anaerobic performance, resulting in significantly lower *V*O_2_ peak and upper and lower limb muscle strength in women than men. Such intrinsic differences, which remained significant even after normalizing data to body weight and composition, demanded to account baseline discrepancy in performance by employing ANCOVA with fitness baseline as covariate, to make homogeneous comparisons. This approach confirmed the different metabolic engagement of the two groups during the trekking, with only women adapting in a significant manner to the short-term physical stress imposed. This finding could be explained by referring to the relative intensity at which the 5-day activity was completed. Physical and physiological demands can be quantified at the individual level considering the relationship between the external and internal workloads (McLaren et al. 2018): the former refers to the physical work being done by the body in the form of movement, while the latter refers to the physiological demands, i.e., the biochemical and biomechanical stress placed on the system. Understanding the relationship between these loads is essential to know the stresses put on the individual during physical activity, which vary considerably. Some activities are homogeneous, being mostly aerobic in nature, while others are more variable, having bouts of higher intensity targeting limb muscles anaerobically followed by periods of aerobic, cardiorespiratory activity, as is the case of hiking and trekking. Therefore, since the trekking was planned and completed as a group-based activity, women were likely performing at a higher intensity than men.

The Selvaggio Blu wild trek here examined was particularly demanding at the lower limb muscular level, due to the total 14,300-m height differential out of a 56-km route to be completed in a relatively short period of time. Given this external load, the internal impact proved higher among women, who also exhibited significantly higher energy expenditure, confirming higher metabolic engagement. Considering the different impact on the two groups, group-based outdoor activities involving both sexes should be tailored on physical and physiological differences to have both women and men adequately challenged and engaged to achieve performance/health effects.

Acute adaptations to the physical activity completed (e.g., reduced fat mass, increased fat-free mass and metabolically active tissue) were somewhat expected, while other findings were less predictable considering the very short duration of the intervention. For instance, significant shifts in the compartmentalization of cellular water and hydration status, found in the present study, are generally expected following longer training programs specifically targeting muscles, such as 12- to 18-week resistance training (Ribeiro et al. 2014; Dos Santos et al. 2016; Souza et al. 2017). What is generally observed following sustained resistive programs is a significant increase in TBW and, more specifically, in ICW at the expense of ECW, according to the “Cell Swelling Theory” as a possible explanatory mechanism (Häussinger et al. 1993). This theory proposes cellular volume as the main signal for the metabolic orientation of cells: an increase in cellular hydration (swelling) acts as an anabolic proliferative signal, whereas cell shrinkage is catabolic and antiproliferative (Häussinger et al. 1993; Lang et al. 1998). Following the trekking event here examined, which specifically targeted the lower limb muscles, women displayed a significant reduction in ECW-to-ICW ratio, at a large effect size. According to the cell swelling theory (Häussinger et al. 1993), the increase in ICW should be interpreted as an anabolic signal to promptly replenish glycogen stores after exercise-induced depletion. Seminal works indicate that 3–4 g of water is bound to each gram of glycogen (Olsson and Saltin 1970) and that muscle is the main reservoir of water in the body (Sawka 1992). In this regard, the significant changes in body composition variables describing skeletal muscle mass, appendicular skeletal muscle mass and body cell mass should be viewed as strictly related to the observed changes in cellular water compartmentalization rather than as a result of muscle protein synthesis (MPS), which is not expected after only few days, although quite intensive, of physical activity. Sustained resistive exercise targeting muscles over several weeks is, in fact, needed to induce cumulative periods of positive net protein balance, which requires that the rate of MPS exceeds the rate of muscle protein breakdown (MacDougall et al. 1995; Phillips et al. 1997). Before MPS-related muscle growth takes place, muscles can appear larger following acute training due to sarcoplasmic hypertrophy. The role of glycosomes during exercise is crucial, as these granules of glycogen have multi-enzymatic activity that promotes, among other metabolic processes, glycogenolysis (Shearer and Graham 2004). When undergoing physical activity, the body experiences metabolic stress, which promotes glycogen release from glycosomes into the sarcoplasm, causing the entire cell to inflate. Subsequently, the whole muscle apparently increases in size as each cell expands. As outlined above, while cell inflation does not coincide with MPS, it leads to increased ICW to counteract proteolysis and stimulate glycogen synthesis and anabolism (Lorenzo et al. 2019). These considerations are specifically pertinent to postmenopausal women and, in general, to the elderly, as age-related intracellular dehydration has been shown to contribute significantly to anabolic resistance, i.e., the inability of anabolic stimuli to prompt MPS (Hooper et al. 2014). Anabolic resistance is considered the main determinant of the age-related loss in muscle mass and strength that is responsible for sarcopenia and functional decline in the elderly (Burd et al. 2013). A short but intensive period of muscle-engaging physical activity like the trekking here examined, would act as anabolic primer by counteracting intracellular dehydration and the associated catabolic cell orientation. However, the relationship between anabolic resistance, intracellular dehydration and sarcopenia is not fully clarified for myocytes, as other key determinants are involved, including the mammalian target of rapamycin pathway, and insulin resistance (Schliess et al. 2006).

Another finding of the study relates to the patterns of utilization of energy substrates to sustain the incremental CPET, as assessed by ergospirometabolimetry based on the respiratory exchange ratio (RER = CO_2_ production/O_2_ consumption) values. Following the trekking period, only women displayed significant changes in fuel utilization, with lower reliance on carbohydrate oxidation to obtain energy, at moderate-to-large effect sizes, suggesting a metabolic shift favoring lipid utilization to sustain the same CPET workloads completed at baseline. The greater reliance on lipids observed among women following the intervention is in line with previous works reporting changes in substrate oxidation towards higher lipid utilization after acute resistive exercise targeting lower limb muscles (Phelain et al. 1997; Binzen et al. 2001). However, this result needs careful interpretation as energy substrate patterns of utilization are better estimated through steady-state stints of aerobic exercise performed at moderate-intensity, generally between 40 and 60% of peak O_2_ uptake (Cano et al. 2022).

It is well established that as exercise intensity increases the contribution of lipids as fuel source declines, resulting in greater reliance on carbohydrate utilization (Cano et al. 2022). Muscle performance during intensive yet submaximal efforts, such as those required for the trek here administered, is heavily dependent on the anaerobic metabolism of stored glycogen as energy source, leading to its fast depletion. When the exercise is discontinued and recovery begins, lipid oxidation becomes the main metabolic pathway to obtain energy whereas carbohydrates are spared to be used for resynthesizing those glycogen stores depleted while exercising. In addition, for the intervention here proposed, participants had to mountaineer both uphill and downhill intermittently for an average of 8 h/day, thus promoting several cycles of glycogen depletion/resynthesis via lipid oxidation. In this context, the intensive, intermittent aerobic/anaerobic exercise specifically involving muscle glycogen may have also contributed to a possible lipid deficit both during the trekking day and in the post-exercise state, which may have led to increased lipid oxidation and an overall change in energy balance. The concurrent, significant reduction in percent body fat (− 20.4%) observed in women seems in line with this hypothesized mechanism. However, while we cannot rule out that this effect merely consisted of a short-lived, acute response, the lack of changes in PETCO_2_ seems to limit the validity of our findings about enhanced fat turnover, warranting confirmation over a larger sample of participants and, importantly, using steady-state exercise protocols rather than the incremental testing here employed to appraise energy substrate patterns of utilization.

### Study limitations

Beyond its preliminary nature and the small sample size, which limits the external validity of our findings and demands careful interpretation, this pilot study is further limited by other non-negligible aspects. Among these, while participants were matched by age, anthropometric characteristics, training status category and mountaineering practice, for logistic and safety reasons the intervention was delivered as a supervised, group-based activity, with all participants completing each of the five stages and the whole route within the same time. This resulted in different relative intensities at which men and women exercised, whereby only women were engaged enough to adapt to the activity. Future studies should examine gender differences not only pairing subjects according to the above criteria but also administering the intervention at matched relative intensities.

Another limitation relates to the use of RER to estimate substrate oxidation and energy expenditure, and to do so through an incremental rather than steady-state aerobic exercise protocol. While all participants were tested by the same physician employing the same procedures, timings and CPET device at a consistent time of the day, the findings about the metabolic shift towards lipid oxidation need to be confirmed also from evidence of change in PETCO_2_ or equivalents of CO_2_ data.

## Conclusions

In postmenopausal women, a 5-day, intensive and physically/technically demanding outdoor trekking activity led to significant and potentially relevant changes in body composition, energy balance and metabolism that are generally attained following quite longer periods of training. In this perspective, short-term, intensive activities may boost metabolic engagement and act as a primer for cellular anabolism, which would be of relevance to postmenopausal women and the elderly as well. These forms of workout yield other health benefits including decreased body fat, increased lean body mass, metabolic engagement, and increased bone mineral density, all being specific and relevant for postmenopausal women and, in general, the elderly.

In addition, while the specific trek route here examined requires physical and technical skills that are not typically found in the average older adult, engaging in outdoor activities, even intense, which involve elements of muscle strength and endurance, balance, dexterity, coordination, physical awareness, and mountaineering skills would represent an appealing and promising way to contrast age-related morphological and functional loss. In this context, provided that all candidates to participate in wild treks are thoroughly screened from a clinical standpoint, trekking in natural, challenging environments may be also viewed as a preventive strategy not only for avoiding the onset of sarcopenic symptoms but also to reduce cardiovascular and mortality risk.

## Data Availability

Data will be made available upon request to the corresponding author.

## Abbreviations

ASMM: Appendicular skeletal muscle mass
BCM: Body cellular mass
BIVA: Bioelectrical impedance vector analysis
BMR: Basal metabolic rate
CHO: Carbohydrates
CI: Confidence interval
CPET: Cardiopulmonary exercise test
ECW: Extracellular water
EE: Energy expenditure
EE%CHO: Energy expenditure derived from carbohydrate oxidation
EE%FAT: Energy expenditure derived from fat oxidation
FFM: Fat-free mass
FM: Fat mass
HR: Heart rate
ICW: Intracellular water
MPS: Muscle protein synthesis
PETCO_2_: Partial pressure of end-tidal carbon dioxide
O_2_: Oxygen
RER: Respiratory exchange ratio
RM-ANOVA: Repeated-measures analysis of variance
RM-ANCOVA: Repeated-measures analysis of covariance
SDs: Standard deviations
SMM: Skeletal muscle mass
TBW: Total body water
*V*′*E*: Pulmonary ventilation
*V*O_2_: Volume of oxygen consumption
*V*O_2_/kg: Volume of oxygen consumption normalized by body weight
Vt1: First ventilatory threshold
Vt2: Second ventilatory threshold
